# Supplementary material for: Identification of ‘erasers’ for lysine crotonylated histone marks using a chemical proteomics approach
Source: eLife. 2014 Nov 4;3:e02999. doi: 10.7554/eLife.02999 (PMC4358366; doi:10.7554/eLife.02999)
Supplement: Supplementary file 1. — Diffraction data and structure refinement statistics. DOI: http://dx.doi.org/10.7554/eLife.02999.022 [file elife02999s002.docx]

**Supplementary file 1.** Diffraction data and structure refinement statistics.

|  | Human_Sirt3-H3K4Cr |
| --- | --- |
| **Data collection** |  |
| Space group | P4_3_2_1_2 |
| Cell dimensions |  |
| *a*, *b*, *c* (Å) | 138.09, 138.09, 225.34 |
| α, β, γ (°) | 90, 90, 90 |
| Resolution (Å) | 50.00-2.95 |
| *R*_sym_ or *R*_merge_ (%) | 9.0 (70.5) |
| *I* / σ*I* | 19.96 (2.93) |
| Completeness (%) | 99.5 (99.8) |
| Redundancy | 7.1(7.0) |
|  |  |
| **Refinement** |  |
| Resolution (Å) | 39.25-2.95 |
| No. reflections | 45642 |
| *R*_work_ / *R*_free_ (%) | 22.45 /27.80 |
| No. of protein residues | 1644 |
| No. of ligand/ion molecules |  |
| Crotonyl H3 K4 | 6 |
| Zn | 6 |
| R.m.s deviations |  |
| Bond lengths (Å) | 0.010 |
| Bond angles (°) | 1.370 |

Values in parentheses are for the highest resolution shell
