# Supplementary material for: Identification of ‘erasers’ for lysine crotonylated histone marks using a chemical proteomics approach
Source: eLife. 2014 Nov 4;3:e02999. doi: 10.7554/eLife.02999 (PMC4358366; doi:10.7554/eLife.02999)
Supplement: Supplementary file 2. — Primers information. DOI: http://dx.doi.org/10.7554/eLife.02999.023 [file elife02999s003.docx]

**Supplementary file 2.** Primers information

| **Gene name** | **Sequence 5'-3'** | **Application** |
| --- | --- | --- |
| *Baz2a* | Forward (F): ATACTTGGCATTATTGCTGCTCTTG | ChIP-qPCR |
|  | Reverse (R): TGTCTTCCTGCTGTTGACCTTTCT |  |
|  | F: CTAAGAGGCGAGCAGGAGACCCTGG | Gene expression analysis |
|  | R: CAGGATCTCGTATCCACCACC |  |
| *Brip1* | F: TCAGAATGTTCTTCTTTGCCTTAACCACA | ChIP-qPCR |
|  | R: GGAGAATATTTTCTGGGTTCACTTGGAAGA |  |
|  | F: AACTCCTTTTCGCCACAGAAACCCC | Gene expression analysis |
|  | R: TGGAACCCCTGAATATGCCGTCCTC |  |
| *Corin* | F: GCTCTAGAGAGTTTTCTGGGGAGCTA | ChIP-qPCR |
|  | R: GGAGGTAGTGAAGTGCCAACATAAGC |  |
|  | F: AGTCTGACGAGGTCAACTGCTCCTG | Gene expression analysis |
|  | R: ATCACTCCCATCCTTGCAGTCCTCG |  |
| *Ptk2* | F: CTTTTCCCCCATCCTTCCCCTCTCC | ChIP-qPCR |
|  | R: CCTGCCCTTAGGCAGTCATAGTGCT |  |
|  | F: TCGGCTTGGCCCTGAGGACATTATT | Gene expression analysis |
|  | R: TCTTGCTGGAGGCTGGTCATGACAT |  |
| *Tshz3* | F: AGAGGGGTGAGACAACAGCAACGAT | ChIP-qPCR |
|  | R: CCCTTGGTAGTCAAAGACATTGCCA |  |
|  | F: CGCAGCAGCCTATGTTTCCGAAGAG | Gene expression analysis |
|  | R: TCACTGATGTGTGACTCGCTGTCCA |  |
| *Wapal* | F: CACCACACAAGGGAAACTTGGGTCC | ChIP-qPCR |
|  | R: AGGTGGCTTCTTAGGATCTGGTTCA |  |
|  | F: TAGTGCTCGGAATCGGCACTGTCTT | Gene expression analysis |
|  | R: GCTCTCGCTCAAGGAATAGCTGCAC |  |
| *Zfat* | F: CCAATGTGCTAAAACAGAGGGCCACA | ChIP-qPCR |
|  | R: TGCTTCAGACTCAGGTGGCTTTCCA |  |
|  | F: AAGTCAAGCAGGCTAGGTCCCACTC | Gene expression analysis |
|  | R: ATAGCTGCACTGGGGGCACTTGTAT |  |
| *Gapdh* | F: CAATTCCCCATCTCAGTCGT | ChIP-qPCR |
|  | R: TAGTAGCCGGGCCCTACTTT |  |
|  | F: GATGACATCAAGAAGGTGGTGAA | Gene expression analysis |
|  | R: GTCTTACTCCTTGGAGGCCATGT |  |
| *Sirt3* | F: TGGAAAGCCTAGTGGAGCTTCTGGG | Gene expression analysis |
|  | R: TGGGGGCAGCCATCATCCTATTTGT |  |
